# The impact of depression at preconception on pregnancy planning and unmet need for contraception in the first postpartum year: a cohort study from rural Malawi

**DOI:** 10.1186/s12978-023-01576-1

**Published:** 2023-02-27

**Authors:** Raquel Catalao, Hilda Chapota, Genesis Chorwe-Sungani, Jennifer Hall

**Affiliations:** 1 King’s College London and South London and the Maudsley NHS Foundation Trust, London, UK; 2 Parent and Child Health Initiative Trust (PACHI) Program, Lilongwe, Malawi; 3 Mental Health at Kamuzu College of Nursing, University of Malawi, Lilongwe, Malawi; 4 UCL Institute for Women’s Health, London, UK

**Keywords:** Depression, CMDs, Contraception, Pregnancy, LMUP, Family planning

## Abstract

**Background:**

The impact of depression on women’s use of contraception and degree of pregnancy planning in low-income settings has been poorly researched. Our study aims to explore if symptoms of depression at preconception are associated with unplanned pregnancy and nonuse of contraception at the point of conception and in the postpartum period.

**Methods:**

Population-based cohort of 4244 pregnant women in rural Malawi were recruited in 2013 and were followed up at 28 days, 6 months and 12 months postpartum. Women were asked about symptoms of depression in the year before pregnancy and assessed for depression symptoms at antenatal interview using the Self‐Reporting Questionnaire‐20, degree of pregnancy planning using the London Measure of Unplanned Pregnancy and use of contraception at conception and the three time points postpartum.

**Results:**

Of the 3986 women who completed the antenatal interview, 553 (13.9%) reported depressive symptoms in the year before pregnancy and 907 (22.8%) showed current high depression symptoms. History of depression in the year before pregnancy was associated with inconsistent use of contraception at the time of conception [adjusted relative risk (adjRR) 1.52; 95% confidence interval (1.24–1.86)] and higher risk of unplanned [adjRR 2.18 (1.73–2.76)] or ambivalent [adj RR 1.75 (1.36–2.26)] pregnancy. At 28 days post-partum it was also associated with no use of contraception despite no desire for a further pregnancy [adjRR 1.49 (1.13–1.97)] as well as reduced use of modern contraceptives [adj RR 0.74 (0.58–0.96)]. These results remained significant after adjusting for socio-demographic factors known to impact on women’s access and use of family planning services, high depression symptoms at antenatal interview as well as disclosure of interpersonal violence. Although directions and magnitudes of effect were similar at six and 12 months, these relationships were not statistically significant.

**Conclusions:**

Depression in the year before pregnancy impacts on women’s use of contraception at conception and in the early postpartum period. This places these women at risk of unplanned pregnancies in this high fertility, high unmet need for contraception cohort of women in rural Malawi. Our results call for higher integration of mental health care into family planning services and for a focus on early postnatal contraception.

**Supplementary Information:**

The online version contains supplementary material available at 10.1186/s12978-023-01576-1.

## Plain language summary

Family planning programmes have traditionally focused on increasing access to modern contraceptive methods. There is growing evidence that merely increasing access will not reach every woman. More focus on improving the quality of the family planning programmes and developing targeted interventions for women and men not currently reached with the current models is necessary. Despite the high prevalence of depression in women of reproductive age living in LMICs, its impact on women’s access and use of contraception has been largely neglected. Our study using data from a cohort of pregnant women recruited in rural Malawi aimed to investigate if depression in the year before pregnancy impacted on women’s risk of having an unintended pregnancy and on contraceptive use at time of pregnancy and in the postpartum period. Our results show that women who reported depression in the year before pregnancy had increased risk of inconsistent contraceptive use and having an unplanned pregnancy. They were also more likely to not use contraception in the early postpartum period and choose less effective methods, with important consequences for risk of subsequent unplanned pregnancies. Our results highlight a need for health services to develop holistic models of care for women where both their mental and reproductive health needs are met.

**Supplementary Information:**

The online version contains supplementary material available at 10.1186/s12978-023-01576-1.

## Introduction

There is a high prevalence of common mental disorders (CMDs; ie anxiety and depression) in women of reproductive age in low and middle income countries (LMICs) [1], yet their impact on reproductive behaviours has been largely neglected. There is evidence from high-income countries that depression is associated with increased rates of unintended pregnancy in young adulthood and shorter pregnancy spacing [2–4]. Depression and stress have also been found to be associated with lower rates of contraceptive use and choice of less effective methods, mostly in clinical samples from North America [5, 6]. There is lack of replicating studies from LMICs but recent evidence showed that high CMD symptoms postpartum were associated with subsequent unmet need of contraception in a sample of women in rural Ethiopia [7].

Most literature on determinants for unmet need for contraception in LMICs has focused on socio-demographic characteristics of the women or factors pertaining to knowledge and access to family planning services [8]. Nevertheless, women often cite concerns about contraceptive side effects or health related reasons for not using contraception as well as lack of regular sexual intercourse [9]. It is estimated that mitigating unmet need for contraception in developing regions could avert over 50 million unintended pregnancies, thereby preventing the deaths of 70,000 women from pregnancy-related causes annually [10]. Despite progress in improving access to contraception, striking inequalities still exist across countries and among vulnerable populations [11]. Acknowledging the challenge, the 2030 Agenda for Sustainable Development includes under Goal 3 to ‘ensure universal access to sexual and reproductive healthcare services, including family planning, by 2030’ [12].

The relationship between poor mental health before pregnancy and subsequent use of contraception has not been investigated in high fertility, high unmet need for contraception settings. For this study, we aimed to investigate if depression is associated with unplanned pregnancy and use of contraception in a cohort of pregnant women in rural Malawi, we hypothesized that symptoms of depression at preconception (here considered to be the year before pregnancy) are associated with a higher risk of unplanned pregnancy and nonuse of contraception at the point of conception and in the postpartum period, independently of other known determinants.

## Methods

We conducted a secondary analysis of a cohort study set in rural Malawi to assess pregnancy intention and its relation to maternal, perinatal and neonatal outcomes [13, 14]. A population-based sample of 4244 pregnant women were recruited in 2013 and were followed up at 28 days, 6 months and 12 months postpartum.

### Setting

This study was conducted in the Mchinji District, a rural district in Malawi, in southern-central Africa, with a population over 530,000, 23% of whom are women of childbearing age (121,950). Around 90% of the population are subsistence farmers. Fertility rates in this region remain high, with a total fertility rate of 6.3 children per woman at the time the study was being conducted, yet the total wanted fertility rate was 4.6 children per woman, and there was a high unmet need for family planning (29.3% in married women) [15]. Family planning services are provided free of charge in Malawi through government health facilities, and are available for purchase through private clinics such as ‘Banja la Mtsogolo’, a Marie Stopes International Partner. On average women live almost 6 km from the nearest health facility; a distance often needed to be covered on foot [13]. Previous research divided Mchinji District into 49 geographical areas; from this sampling frame a random sample of 25 areas were selected.

### Participants

Eligible participants were all pregnant women aged 15 and over living within the district demographic surveillance areas selected between March and December 2013 [14]. Women were eligible to participate at any point during their pregnancy and were interviewed at home by trained data collectors, after giving informed consent, using a questionnaire programmed using CommCareODK software on a smartphone. 4244 pregnant women between two and nine months pregnant (median six, mean 5.98) completed the antenatal interview; 3986 (93.9%) were followed up at 28 days postpartum. This was a rolling cohort and was stopped when the last recruited woman reached the 28-day postpartum point. As women were recruited at different gestations, some women, but not all, were eligible and completed interviews at 6 months and 12 months postpartum.

### Measures

#### Depression

In the absence of a locally validated tool, we ascertained history of depression in the year before pregnancy by asking women at the antenatal interview if they had experienced 2 weeks or more of low mood or two weeks of more of anhedonia. A positive reply to any of these questions was defined as history of depression in the year preconception. Postnatal depression was assessed using the validated Chichewa version of the World Health Organization’s 20-question screening tool, the Self-Reporting Questionnaire 20 (SRQ 20). The SRQ 20 was previously found to be valid (Sensitivity = 76.3%, Specificity = 81.3%) and reliable (Cronbach’s α = 0.83) instrument for screening perinatal depression in Malawi [16]. In this study, a cut off score of ≥ 8 was used to determine depression before pregnancy [17].

#### Degree of pregnancy planning

The degree of intention of women’s current pregnancy was assessed using the London Measure of Unplanned Pregnancy (LMUP) at antenatal interview. By asking six questions, each scored zero, one or two, the LMUP scores pregnancy intention on a continuous scale from zero to 12 with each increase in score representing an increase in the degree of pregnancy intention [18]. The LMUP was validated for use in the Chichewa language in Malawi prior to the establishment of this cohort and found to be a valid and reliable measure of pregnancy intention in this setting [19]. Women’s scores were grouped into three categories unplanned, ambivalent and planned.

#### Use of contraception

At the antenatal questionnaire women were asked if in the month they became pregnant they were using contraception and how regularly. Postnatally women were asked if they were using family planning methods and which methods were being used. Contraceptive methods were classified as modern if they were products or medical procedure that interfere with reproduction from acts of sexual intercourse, therefore condoms, oral contraceptive pills, tubal ligation (postnatally), coil, injectable contraceptives and implants were classified as modern methods. Abstinence and withdrawal methods were considered traditional. Lactation amenorrhea (LAM) was considered traditional as although is a very effective method if practiced correctly, it is well acknowledged that often only a minority of women who report breastfeeding as a method of contraception meet the correct-practice criteria for LAM and this method is not considered effective after the early postpartum period [20].

### Confounders

Socio-economic and demographic factors known to impact on women’s unmet need for contraception were selected from available literature [21]. These included maternal age, education, marital status, distance to the health facility, socio-economic status, parity and religion. A principal components analysis (PCA) was conducted to generate an asset-based measure of socio-economic status (SES). In addition to ownership of assets such as a bicycle and radio, variables included in the PCA were household characteristics, such as floor and roof materials, household density, and access to water and sanitation facilities [14]. GPS readings of the location of the interview were taken and were used to calculate the distance to the nearest health facility, ‘as the crow flies’. Interpersonal Violence (IPV) was assessed using the Abuse Assessment Screen [22]. This asks about experience of abuse ever, in the last year or while pregnant as well as experience of sexual abuse.

### Statistical analyses

Stata version 15 software was used for data analysis [23]. Our analysis strategy was hypothesis-driven, using multinominal logistic regression to investigate the association between depression in the preconception year and use of contraception at conception; in early postpartum (28 days) and late postpartum period (at 6 and 12 months). Women who reported to be planning a pregnancy or no current partner were excluded from the analysis. These hypotheses were partially and fully adjusted for known confounders identified a priori*,* including demographic and socio-economic characteristics. The analysis of the relationship between depression at preconception and unmet need for contraception at 28 days postpartum was adjusted for high depression symptoms at pregnancy (SRQ score >  = 8) and the analysis for the relationship between depression at preconception and unmet need for contraception at 6 months was adjusted for high depression symptoms at pregnancy as well as in the early postpartum. Missing data on use of contraception (< 8% of total sample at 6 months) was addressed by case wise deletion at the analysis stage.

## Results

### Loss of follow up

The recruitment process for this cohort and baseline characteristics have been described elsewhere [13, 14], but in brief, of the 5887 pregnant women identified, 4244 completed the antenatal interview. The majority of the women not interviewed, 71%, had already delivered when visited and were no longer eligible; less than 1% of eligible women declined to participate. Women were aged 15–49 (median 24), the vast majority (over 90%) were married and had no education or primary education only (86%). Most women were Christian and were from the Chewa tribe. Women reported up to 15 previous pregnancies (median 3) and 12 previous births (median 2).

**Fig. 1:**
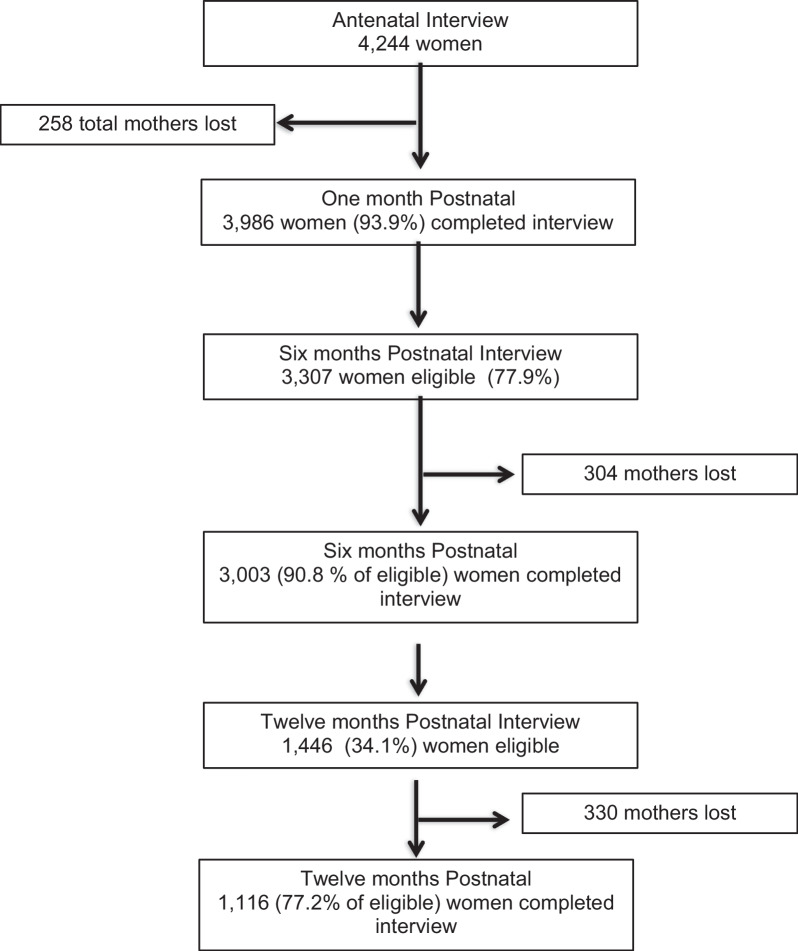
Flow diagram of participants

At 28 days postpartum, 3986 women (93.9% of eligible) completed the interview, a loss of 6.08% mostly due to migration. Of the 3307 women eligible for interview at 6 months postpartum, 3003 (90.8%) were interviewed. 1446 women were eligible for interview at 12 month postpartum of which 1116 (77.2%) completed the questionnaire (Fig. 1).

At 12 months women were not statistically significantly different with regards to age, marital status, education, socio-economic status, parity or history of depression at preconception to those lost at follow up. They were slightly more likely to have experienced abuse in the year before pregnancy (7.1% vs 9.1% p < 0.05) (Additional file 1: Table S1).

At the antenatal interview, 553 women (13.9%) reported a history of depression in the year before pregnancy and 907 women (22.8%) screened positive for high depression symptoms whilst pregnant. The prevalence of high depression symptoms was much lower at 28 days (7.2%) and at 6 months (2.6%) postpartum (Table 1).

**Table 1:** Prevalence of depression symptoms and contraceptive use among participants

	Year before conception (N = 3986)	During pregnancy (N = 3986)	28 days postpartum (N = 3976)	6 months postpartum (N = 2776)
Depression symptoms	553 (13.9)	907 (22.8)	285 (7.2)	72 (2.6)
Contraceptive use (modern and traditional methods)	1339 (33.6)	–	3069 (83.5)	2381 (86.9)

Data = n(%)

*Women were asked if they experienced two or more weeks of low mood or anhedonia in the year before pregnancy

**SRQ >  = 8 – high depression symptoms

### Depression in the year before conception, degree of pregnancy planning and contraceptive use

Whereas the majority of women [n = 2643 (66.4%)] reported not using contraception at the time they conceived, 28.1% (n = 1121) reported they were occasionally using contraception or knew the method had failed, and only 5.5% reported consistent use at the time of conception (Table 2).

**Table 2:** Depression in the year before pregnancy, degree of pregnancy planning at conception and contraceptive use during cohort period

	Depression in the year preconception			
	Yes	No	Total	
*Degree of pregnancy planning (LMUP score)*				
Planned	152 (27.5%)	1620 (47.2%)	1772 (44.5%)	X^2^ (2, N = 3986) = 82.2, p < 0.001
Ambivalent	145 (26.2%)	775 (22.6%)	920 (23.1%)	
Unplanned	256 (46.3%)	1038 (30.2%)	1294 (32.5%)	
*At the time became pregnant*				
Always used contraception	34 (6.2%)	184 (5.2%)	218 (5.5%)	X^2^ (2, N = 3982) = 29.8, p < 0.001
Sometimes used/ knew method failed	207 (37.4%)	914 (26.7%)	1121 (28.1%)	
Not using contraception	312 (56.4%)	2331 (68.1%)	2643 (66.4%)	
Total	553 (13.9%)	3422 (86.1%)	3982 (100%)	
*Contraception 28 days postpartum*				
Not using FP and does not want to get pregnant	96 (19.5%)	398 (13.0%)	490 (13.8%)	X^2^ (2, N = 3559) = 27.2, p < 0.001
Traditional method	290 (60.0%)	1720 (56.0%)	2010 (56.5%)	
Modern method	106 (21.5%)	953 (31.0%)	1059 (29.7%)	
Total	492 (13.8%)	3067 (85.2%)	3559 (100%)	
*Contraception 6 months postpartum*				
Not using FP and does not want to get pregnant	56 (15.4%)	241 (10.4%)	297 (11.1%)	X^2^ (2, N = 2678) = 9.2, p = 0.01
Traditional method	96 (26.5%)	585 (25.3%)	681(25.4%)	
Modern method	211 (58.1%)	1489 (64.3%)	1700 (63.5%)	
Total	363 (13.6%)	2315 (86.4%)	2678 (100%)	
*Contraception 12 months postpartum*				
Not using FP and does not want to get pregnant	14 (9.8%)	55 (7.0%)	69 (7.4%)	X^2^ (2, N = 932) = 3.4, p = 0.185
Traditional method	31 (21.7%)	137 (17.4%)	168 (18.0%)	
Modern method	98 (68.5%)	597 (75.6%)	695 (74.6%)	
Total	143 (15.3%)	789 (84.7%)	932 (100%)	

Women with depression in the year before conception were more likely to report occasional contraception use or method failure at the time they became pregnant in both the unadjusted and fully adjusted models [adj RR 1.52 (95%CI 1.24–1.86)] (Table 3). There were significant differences in the degree of pregnancy planning between women with history of depression in the year before conception and those without. Women with history of depression were more likely to report the pregnancy was unplanned [n = 256 (46.3%) vs n = 1038 (30.2%)] or ambivalent [n = 145 (26.2%) vs n = 775 (22.6%)] (Table 2). This increased risk of unplanned pregnancy remained significant after adjusting for socio-demographic factors such as maternal education, asset index and IPV in past year [adj RR 2.18 (1.73–2.76)] (Table 3).

**Table 3:** Multinomial logistic regression for women with history of depression in the year preceding pregnancy and degree of planning of current pregnancy

	RRR	95% CI	P value
*Planned pregnancy (base)*			
Unplanned pregnancy	2.63	2.12–3.26	≤ 0.001
Ambivalent	1.99	1.56–2.54	≤ 0.001
*Adjusted model for maternal age, education, marital status, distance to health facilities, asset index, parity, religion, IPV in past year*			
Unplanned pregnancy	2.18	1.73–2.76	≤ 0.001
Ambivalent	1.75	1.36–2.26	≤ 0.001
*Not using contraception when became pregnant (base)*			
Always used contraception	1.38	0.94–2.03	0.1
Sometimes used contraception/ knew method failed	1.69	1.40–2.05	≤ 0.001
*Adjusted model for maternal age, education, marital status, distance to health facilities, asset index, parity, religion, IPV in past year*			
Always used contraception	1.21	0.81–1.82	0.3
Sometimes used contraception/ knew method failed	1.52	1.24–1.86	≤ 0.001

In the early postpartum period over half of the women [n = 2010 (56.5%)] were using traditional contraceptive methods including lactation amenorrhea whereas 490 women (13.8%) reported not using any contraceptive method despite no wish to become pregnant again at that time (Table 2). Women with depression in the preconception period were significantly more likely to not be using contraception [adj RR 1.50 CI (1.14–1.97)] and less likely to be using modern methods [adjRR 0.74 (0.58–0.94)] in the unadjusted, adjusted for depressive symptoms in pregnancy and fully adjusted model (Table 4).

**Table 4:** Multinomial logistic regression of use of contraception postpartum and depression in the year preceding pregnancy for women who do not currently desire another pregnancy

	Early postpartum (28 days) n = 3559		6 months postpartum n = 2678		12 months postpartum n = 932	
	RRR	95% CI	RRR	95%CI	RRR	95% CI
*Using traditional contraceptive (baseline)*						
Not using FP but don’t want to get pregnant	1.45	1.12–1.87	1.42	0.98–2.03	1.13	0.56–2.27
Using modern contraceptive method	0.66	0.52–0.83	0.86	0.67–1.12	0.72	0.47–1.13
*Adjusted model for depression in pregnancy (SRQ > 8)*						
Not using FP but don’t want to get pregnant	1.42	1.09–1.86	1.27	0.87–1.85	0.89	0.42–1.88
Using modern contraceptive method	0.72	0.56–0.92	0.83	0.64–2.09	0.69	0.43–1.11
*Adjusted model for depression in pregnancy and postpartum, maternal age, education, marital status, distance to health facilities, asset index, religion, parity and abuse in the past year*						
Not using FP but don’t want to get pregnant	1.50	1.14–1.97	1.18	0.79–1.75	0.83	0.38–1.84
Using modern contraceptive method	0.74	0.58–0.95	0.84	0.63–1.11	0.70	0.42–1.14

At 6 months the majority of the women were using modern methods of contraception (n = 1700, 63.5%) however 25.4% were using traditional methods and 11.1% were not using any contraceptive method despite no wish to become pregnant. Women with a history of depression in the year before pregnancy were more likely to not be using contraception despite not wanting to get pregnant [RR 1.42 CI (0.98–2.03)] however the strength of this association was not significant (Table 4) and factors such as marital status, maternal education level and distance from the health facilities seem to be more significant in determining use of modern contraception.

At 12 months 18.0% and 7.4% of women interviewed were using traditional methods or no method at all, respectively. However, there were no significant differences between women with history of depression in preconception period and those without, this may be partly due to smaller sample size at these time points.

## Discussion

In our study, depression symptoms were most prevalent before and during pregnancy with around one fifth of women reporting high level of symptoms at antenatal interview but significantly lower numbers reporting symptoms at later stages. We found that a self-reported history of depression in the year before pregnancy is associated with inconsistent use of contraception at the time of conception, higher risk of unplanned and ambivalent pregnancies, no use of contraception despite no desire for a further pregnancy in the early postpartum period as well as reduced use of modern contraceptive method at 28 day post-partum. These results remained significant after adjusting for socio-demographic factors known to impact on women’s access to and use of family planning and depression symptoms in pregnancy. Although the point estimates were similar and in the same direction at 6 months and 12 months, the differences were not significant.

In our cohort less than a third of women were using modern contraceptive methods in the early postpartum with the majority relying on lactational amenorrhea as their main contraceptive method. Whereas the number using modern methods increased in subsequent months, to over two thirds using modern contraceptive methods at 12 months, a significant proportion still relied on traditional methods and reported no contraception use despite no desire for a further pregnancy at this time point. Our findings show that women with a history of depression in the year before pregnancy are at increased risk of a subsequent unplanned pregnancy.

Our study adds to the growing literature on unmet need for contraception amongst women with mental health disorders. Studies from high income countries and mostly from clinic-based samples have previously highlighted an association between depression and stress and contraceptive non-use and reduced odds of consistent contraceptive use, especially when using withdrawal, condoms, and birth control pills compared to women without symptoms [5, 6, 24]. A recent study using primary care data linked with obstetrics and gynecology records in the US has also reported that women with mental health disorders have increased odds of Long-Acting Reversible Contraception removal [25]. In those settings, suggested mechanisms underlying this association are that psychological distress may negatively impact the decision making and risk assessment abilities of women with regards to contraceptive behavior [26, 27]. An alternative possible mechanism is that women with depression may be more susceptible to perceive somatic side effects of hormonal contraception and therefore discontinue use more readily [28]. Globally, side effects are the most common reason for women to discontinue contraception [29] however no studies exist investigating if women with depression experience find it harder to tolerate side effects and therefore discontinue more readily. IPV has also been shown to be associated with a reduction in women’s use of contraception [30] and could be an important confounder, however our results remained significant after adjusting for disclosure of abuse in past year. The vast majority of women in our cohort were Christian and the religious recommendation in this setting is to return to sexual activity following childbirth. There may be differences in how women adopt contraceptive methods depending on their religious beliefs but this could not be explored in this study.

Whereas much research has recently focused on the hormonal contraception effects on mood with no consistent results [31] and a large body of evidence supports an association between unintended pregnancies and perinatal depression [32], little is known about how depression may impact on use and choice of contraceptive methods, particularly in low income settings. A recent study from rural Ethiopia reported that women with high depression symptoms in the late postpartum have higher unmet need for contraception [7]. Similarly to our study the relationship seemed to have a temporal element, with a relationship seen between high depression symptoms at 12 months and unmet need for contraception at 2.5 years but not at 3.5 years. Variations in the temporal gradients may be partly explained by the timings when women are more likely to experience depression symptoms. Whereas in this study from Malawi women had a higher prevalence of depression symptoms during pregnancy than at any other times during the cohort, in the Ethiopian study women showed higher rates of depression symptoms in the early postpartum period. Contextual differences in how women access social support and experience adversity in different parts of their reproductive life may play a role in the prevalence of depression and further studies are required to explore potential mechanisms.

To our knowledge this is the first study to investigate the role of depression before pregnancy on contraceptive use at conception and in the early postpartum, in a low income setting. Our results have important implications as traditionally family planning programmes have focused on increasing access to modern contraceptive methods with little consideration how different groups of women such as adolescents [33], or women experiencing psychological distress may benefit from more targeted interventions in order to have their reproductive rights met. There is growing evidence that merely increasing access will not reach every woman and focus on improving the quality of the family planning programs and develop targeted interventions for women not currently reached with the current models are necessary [29]. Our results also highlight the need for general services, including family planning services, to integrate mental health care into all general health care services as per ambition of the World Health Organization mental health Gap Action Programme [34], Common mental disorders are prevalent in women of reproductive age in LMICs [1] and failing to address them may have long term impact on women’s health, choices and opportunities.

Strengths of our study include the large, representative sample of women from a rural setting in a low-income country, the use of locally validated measures of pregnancy intention and of depression in the pregnancy and the postpartum period and the prospective design with high rates of follow-up. Our study also has several potential limitations. It relies on self-reported data for symptoms of depression in the year before conception and use of contraception and may be subjected to reporting bias. We were also unable to explore the reasons behind contraceptive choices. Whereas we collected data on pregnancy intentions we lacked information on side effects. We can also not exclude that other confounders not used in our models may play a significant role in our hypotheses.

## Conclusion

Depression in the year before pregnancy impacts on women’s risk of unintended pregnancy and use of contraception at conception and early postpartum in this high fertility high unmet need for contraception cohort of women in rural Malawi. Further studies are required to explore the mechanisms by which poor mental health impact on women’s reproductive behaviours in this setting and what interventions can empower women to recover and make informed choices regarding their health and fertility.

## Supplementary Information

**Additional file 1: Table S1.** Socio-demographic characteristics of women completing the antenatal interview.

## Data Availability

All data will be made available from the UCL Discovery database linked to the publication record in the UCL Research Publication Service.

## Abbreviations

Adj: Adjusted
CMD: Common mental disorder
CI: Confidence interval
IPV: Interpersonal violence
LAM: Lactation amenorrhea
LMICs: Low and middle income countries
LMUP: London measure of unplanned pregnancy
RR: Relative risk
SES: Socio-economic status
SRQ 20: Self-reporting questionnaire 20
US: United States

## References

[1] Fisher J, Cabral de Mello M, Patel V, Rahman A, Tran T, Holton S (2012). Prevalence and determinants of common perinatal mental disorders in women in low- and lower-middle-income countries: a systematic review. Bull World Health Organ.

[2] Corcoran J (2016). Teenage pregnancy and mental health. Societies.

[3] Hall KS, Kusunoki Y, Gatny H, Barber J. The risk of unintended pregnancy among young women with mental health symptoms. Soc Sci Med. 2014;0:62. 10.1016/j.socscimed.2013.10.037 PMC3898511 24444840

[4] Patchen L, Caruso D, Lanzi RG (2009). Poor maternal mental health and trauma as risk factors for a short interpregnancy interval among adolescent mothers. J Psychiatr Ment Health Nurs.

[5] Hall KS, Moreau C, Trussell J, Barber J (2013). Role of young women’s depression and stress symptoms in their weekly use and nonuse of contraceptive methods. J Adolesc Health.

[6] Garbers S, Correa N, Tobier N, Blust S, Chiasson MA (2010). Association between symptoms of depression and contraceptive method choices among low-income women at urban reproductive health centers. Matern Child Health J.

[7] Catalao R, Medhin G, Alem A, Dewey M, Prince M, Hanlon C (2020). Mental health impact on the unmet need for family planning and fertility rate in rural Ethiopia: a population-based cohort study. Epidemiol Psychiatr Sci.

[8] Wulifan JK, Brenner S, Jahn A, De Allegri M (2016). A scoping review on determinants of unmet need for family planning among women of reproductive age in low and middle income countries. BMC Womens Health.

[9] Sedgh G, Ashford LS, Hussain R. Unmet need for contraception in developing countries: examining women’s reasons for not using a method. 2016. https://www.guttmacher.org/report/unmet-need-for-contraception-in-developing-countries.

[10] Singh S, Darroch JE, Ashford LS. Adding it up: the costs and benefits of investing in sexual and reproductive health 2014. United Nations Popul Fund. 2014; https://www.guttmacher.org/report/adding-it-costs-and-benefits-investing-sexual-and-reproductive-health-2014.

[11] Kantorová V, Wheldon MC, Ueffing P, Dasgupta ANZ (2020). Estimating progress towards meeting women’s contraceptive needs in 185 countries: a Bayesian hierarchical modelling study. PLOS Med.

[12] UN. United Nations Transforming Our World: the 2030 Agenda for Sustainable Development. A/RES/70/1. United Nations 2015.

[13] Hall Id JA, Barrett G, Copas A, Phiri T, Malata A, Stephenson J (2018). Reassessing pregnancy intention and its relation to maternal, perinatal and neonatal outcomes in a low-income setting: a cohort study. PLoS ONE.

[14] Hall JA, Barrett G, Phiri T, Copas A, Malata A, Stephenson J (2016). Prevalence and determinants of unintended pregnancy in Mchinji District, Malawi; using a conceptual hierarchy to inform analysis. PLoS ONE.

[15] Statistical Office N, Macro I. Malawi 2010 Demographic and Health Survey. http://www.measuredhs.com.

[16] Stewart RC, Kauye F, Umar E, Vokhiwa M, Bunn J, Fitzgerald M (2009). Validation of a Chichewa version of the self-reporting questionnaire (SRQ) as a brief screening measure for maternal depressive disorder in Malawi, Africa. J Affect Disord.

[17] Kumbhar UT, Dhumale GB, Kumbhar UP (2012). Self reporting questionnaire as a tool to diagnose psychiatric morbidity. Natl J Med Res.

[18] Barrett G, Smith SC, Wellings K (2004). Conceptualisation, development, and evaluation of a measure of unplanned pregnancy. J Epidemiol Community Health.

[19] Hall J, Barrett G, Mbwana N, Copas A, Malata A, Stephenson J (2013). Understanding pregnancy planning in a low-income country setting: validation of the London measure of unplanned pregnancy in Malawi. BMC Pregnancy Childbirth.

[20] Festin MPR, Kiarie J, Solo J, Spieler J, Malarcher S, Van Look PFA (2016). Moving towards the goals of FP2020— classifying contraceptives. Contraception.

[21] Nkoka O, Mphande WM, Ntenda PAM, Milanzi EB, Kanje V, Guo SJG (2020). Multilevel analysis of factors associated with unmet need for family planning among Malawian women. BMC Public Health.

[22] Rabin RF, Jennings JM, Campbell JC, Bair-Merritt MH (2009). Intimate partner violence screening tools: a systematic review. Am J Prevent Med..

[23] Statacorp. StataCorp. Stata Statistical Software: Release 15. College Station, TX: StataCorp LLC; 2017.

[24] Stidham Hall K, Moreau C, Trussell J, Barber J (2013). Young women’s consistency of contraceptive use—does depression or stress matter?. Contraception.

[25] Bello JK, Salas J, Meyer D, Heiden-Rootes K, Davis DM, Keegan Garrett E (2020). Mental health diagnoses and early removal of long-acting reversible contraception. J Affect Disord.

[26] Hall KS (2012). The Health Belief Model can guide modern contraceptive behavior research and practice. J Midwifery Womens Health.

[27] Yuen KSL, Lee TMC (2003). Could mood state affect risk-taking decisions?. J Affect Disord.

[28] Hall KS, White KO, Rickert VI, Reame N, Westhoff C (2012). Influence of depressed mood and psychological stress symptoms on perceived oral contraceptive side effects and discontinuation in young minority women. Contraception.

[29] Castle S, Askew I. Contraceptive discontinuation: reasons, challenges, and solutions. London; 2015.

[30] Maxwell L, Devries K, Zionts D, Alhusen JL, Campbell J (2015). Estimating the effect of intimate partner violence on women’s use of contraception: a systematic review and meta-analysis. PLoS ONE.

[31] Robakis T, Williams KE, Nutkiewicz L, Rasgon NL (2019). Hormonal contraceptives and mood: review of the literature and implications for future research. Curr Psychiatry Rep.

[32] Abajobir AA, Maravilla JC, Alati R, Najman JM (2016). A systematic review and meta-analysis of the association between unintended pregnancy and perinatal depression. J Affect Disord.

[33] Li Z, Patton G, Sabet F, Zhou Z, Subramanian SV, Lu C (2020). Contraceptive use in adolescent girls and adult women in low- and middle-income countries. JAMA Netw open.

[34] WHO. Scaling up care for mental, neurological, and substance use disorders. 2008. http://www.who.int/mental_health/mhgap_final_english.pdf. Accessed 8 Mar 2021. 26290926

