# Supplementary material for: The impact of depression at preconception on pregnancy planning and unmet need for contraception in the first postpartum year: a cohort study from rural Malawi
Source: Reprod Health. 2023 Feb 27;20:36. doi: 10.1186/s12978-023-01576-1 (PMC9972717; doi:10.1186/s12978-023-01576-1)
Supplement: Supplementary file 1 — Additional file 1: Table S1. Socio-demographic characteristics of women completing the antenatal interview. [file 12978_2023_1576_MOESM1_ESM.docx]

|  | At baseline  n=3,986 women | | 12 months postpartum  n=954 |
| --- | --- | --- | --- |
| **Maternal Age at start of cohort** | Frequency | Percentage |  |
| 15-19 | 954 | 23.9 |  |
| 20-24 | 1,136 | 28.5 |  |
| 25-29 | 895 | 22.5 |  |
| 30-34 | 588 | 14.8 |  |
| 35-39 | 296 | 7.4 |  |
| 40-49 | 117 | 2.9 |  |
| Mean (SD) | 25.1 (6.6) | | 25.3 (6.6) |
| **Maternal Education** |  |  |  |
| None | 396 | 9.9 |  |
| Primary | 3,018 | 75.7 |  |
| Secondary or above | 572 | 14.4 |  |
| Mean years (SD) | 5.4 (3.12) | | 5.2 (3.16) |
| **Marital status** |  |  |  |
| Married | 3665 | 92.0 |  |
| Unmarried | 321 | 8.0 |  |
| **Parity** |  |  |  |
| 0 | 1145 | 28.7 |  |
| 1-2 | 1337 | 33.5 |  |
| 3+ | 1,504 | 37.7 |  |
| Mean (SD) | 2.2 (2.19) | | 2.2 (2.19) |
| **Distance to health facility** |  |  |  |
| <2.5 kms | 526 | 13.1 |  |
| 2.5-4.99 kms | 923 | 23.2 |  |
| 5.0-7.49 kms | 1,340 | 33.7 |  |
| >7.5 kms | 1,192 | 29.9 |  |
| Mean | 5.9 (3.02) | | 6.1 (3.05) |
| **Religion** |  |  |  |
| Christian | 1,936 | 48.6 |  |
| Catholic | 1,897 | 47.6 |  |
| Muslim | 84 | 2.1 |  |
| Other | 69 | 1.7 |  |
| **Asset index mean (IQR)** | 0.05 (-1.24 / 1.00) | | -0.10 (-1.26/0.76) |
| Abuse in past year | 283 | 7.1 | 87 (9.1%) * |
| Abuse while pregnant | 151 | 3.8 | 42 (4.4%) |

**Table S1. Socio-demographic characteristics of women completing the antenatal interview.**

* p<0.05
